# Virtual Reality as a Preparatory Educational Tool in Non-VR Trauma Simulation for Medical Students: A Qualitative Content Analysis

**DOI:** 10.1007/s40670-026-02822-4

**Published:** 2026-07-17

**Authors:** L. Gyllencreutz, M. Byström, K. Jonsson, M M. Haapamäki

**Affiliations:** 1 Department of Diagnostics and Intervention, Umeå University, Umeå, 901 85 Sweden; 2 Department of Nursing, Umeå University, Umeå, 901 87 Sweden

**Keywords:** Medical students, Simulation-based medical education, Virtual reality, Non-VR trauma simulation

## Abstract

**Background:**

Simulation-based medical education (SMBE) is a well-established and effective method for clinical training, yet its implementation is often resource-intensive and logistically demanding. Virtual reality (VR) has emerged as a promising educational tool, offering immersive, multisensory simulations that may support both procedural and cognitive skill development. In educational context where students must prepare for complex scenarios, VR may serve as a preparatory educational tool. Thus, exploring such simulation formats is well justified to enhance training outcomes.

**Aim:**

To explore medical students’ experiences of using VR as preparation for a subsequent non-VR trauma simulation.

**Methods:**

Thirty-two medical students in their seventh semester who were assigned to use a VR application prior to a scheduled non-VR trauma simulation, participated in this study. Eight focus-group interviews were conducted immediately after the trauma simulation. Data were analysed using qualitative content analysis.

**Results:**

The findings were categorised into four categories: *Perceived educational effect*, *Practical user experience*, *Comparison of VR and other educational forms*, and *Areas for improvement in the VR application*. The students described the VR application as an engaging and motivating learning method that supported autonomous preparation. However, the VR application was not perceived as sufficient to replace traditional educational formats. Key limitations included low interactivity, limited realism, and lack of cognitive flexibility. The participants proposed improvements, such as dynamic patient responses, expanded roles, and increased complexity in order to better reflect clinical practice.

**Conclusions:**

The VR application shows potential as a flexible and accessible preparatory complement to SMBE. However, in its current form, it was not perceived as a suitable standalone learning method. The identified limitations relate to the design and scope of the specific VR application studied rather than to inherent limitations of VR technology. Student perspectives offer important insights into how preparatory VR may support motivation, early cognitive rehearsal and familiarization with simulation workflows, and should be actively considered in the development of VR-based educational tools.

## Introduction

Physical trauma such as blunt and penetrating injuries, represents one of the leading causes of mortality in developed countries, and is frequently associated with long-term disability [1]. Its societal consequences include both immediate and sustained healthcare costs, as well as the loss of physically capable individuals from the workforce [1]. Despite its severity, physical trauma accounts for only a small proportion of annual visits to Swedish emergency departments, meaning limited practical exposure for healthcare personnel [2–4].

Medical education research seeks to identify tools that are efficient, effective, and economically sustainable, with an emphasis on educationally sound, scalable, and adaptable methods suited to evolving clinical contexts [5]. Traditional approaches such as clinical rotations remain central, and have demonstrated educational value [6]. In recent years, simulation-based medical education (SBME) has become a well-established complement, and is widely implemented in undergraduate curricula [6–8]. SBME enables students to practise both technical and non-technical skills in a safe, controlled, and realistic environment [6–9]. However, despite its educational benefits, SBME is often considered resource-intensive and logistically demanding [5–7, 9]. This has prompted interest in alternative, less resource-demanding strategies that can still support effective simulation-based learning.

Virtual reality (VR) is an emerging educational technology with the potential to offer immersive, repeatable, interactive, and standardized training experiences and enhance learning outcomes [10–15]. By leveraging three-dimensional (3D) multimedia and multisensory input, VR facilitates first-person active learning, allowing users to interact with virtual objects and avatars in dynamic environments [14, 16]. This approach supports both procedural training and the development of cognitive competencies within healthcare education, all within a safe and controlled setting [13, 14]. Despite its promise, VR remains a relatively novel tool, and empirical evidence regarding its effectiveness, particularly in clinical and trauma-related training contexts, is still limited, and learning advantages appear to depend on specific perceptual and design factors rather than immersion alone [14, 15, 17].

Existing research on VR in medical education has primarily focused on its impact on knowledge acquisition, skill development, and transferability, which are often assessed through performance tests, questionnaires, and video analysis [14, 18]. However, methodological inconsistencies across studies limit the generalizability of conclusions, highlighting the need for further empirical data to address current knowledge gaps [14]. A key question is whether VR’s enhanced realism and spatial fidelity, enabled by its immersive 3D nature, can improve medical students’ preparedness for managing trauma scenarios [14].

To inform evidence-based educational practice, additional research is needed to evaluate the educational value of VR in undergraduate medical education. The current lack of robust evidence regarding its effectiveness in supporting student learning calls for new approaches. The students’ basic attitude towards VR, their prior understanding of and their own experiences of VR in education form the basis for their acceptance (or dislike) of the use of VR as a learning method. We assume that broad acceptance and the students’ own good experiences of VR are required for a successful broad introduction to an educational programme. To explore these conditions, we wanted to study how students perceive and value training with our application TraumaSim in VR. As such, the aim of this study was to explore medical students’ experiences of VR as preparation for a non-VR trauma simulation within a qualitative study, focusing on the students’ reflections and experiences with VR. This was intended to provide valuable insights and contribute to a more nuanced understanding of the educational potential of VR.

## Materials and Methods

### Participant Recruitment

This study is part of a larger project titled *Virtual Reality Simulation in Clinical Teaching* (VRSinCT), which includes a randomized controlled trial. The primary aim of the VRSinCT project is to investigate whether the combination of brief written materials and 3D VR simulation improves learning outcomes in emergency care, compared to traditional theoretical instruction using the written materials alone.

Within the VRSinCT project, participating seventh semester medical students were randomized into two groups: an intervention group and a control group. In this study, only the intervention group (*n* = 32) participated as the aim was to focus on the students’ reflections and experiences with VR as preparation for providing the non-VR trauma simulation sessions. The intervention group received access to VR equipment, including head-mounted displays, hand controllers, and written preparatory materials. The VR application was designed to support learning in trauma care and was made available approximately one week prior to the scheduled trauma simulation sessions at a Clinical Training Centre (CTC), hereby referred to as the CTC trauma simulation. The students were instructed to engage with the study materials, VR application and written instruction of trauma care (10 pages) for a minimum of two hours.

The CTC trauma simulation was formed part of the curriculum of the seventh semester, including surgery and anesthesiology, of the in total 12 semester long medical program. Each trauma simulation involved three to five students who worked collaboratively to manage critically ill or injured patients in a simulated emergency-room setting, using the structured Airway, Breathing, Circulation, Disability, Exposure (ABCDE) approach. The trauma simulation session aimed to train leadership, decision-making, and clinical management in acute care scenarios. The students’ prior experience with VR headsets varied from none to a few with relatively extensive gaming-related VR use.

### VR Technology

The 3D VR technology used in the VRSinCT project was the Meta Quest 3 system, consisting of a head-mounted display and two handheld controllers. The learning content was delivered through *TraumaSim VR*, an application that simulates an existing trauma room at the Umeå University Hospital. The simulation focused on the initial 20 min following the receipt of a prehospital report from ambulance personnel regarding a critically injured patient.

Within the VR application, the user assumes the role of a trauma team leader, and interacts with virtual avatars representing healthcare professionals, such as an attending emergency department physician and nurses. Three distinct patient scenarios are available, each designed to be completed individually at a time and location that is convenient for the student. To support independent use, an introductory video explaining the training process and functionality of the VR system was also provided, along with a short written manual.

### Design

A qualitative study design based on focus-group interviews was employed.

### Data Collection

The results of this study are based on eight focus-group interviews, each consisting of four medical students from the intervention group. The interviews were conducted in Swedish and immediately following the CTC trauma simulation. The focus-group methodology was chosen to facilitate dynamic discussions that captured both individual perspectives and shared experiences, thereby generating a broader range of insights [19].

The interviews lasted between 18 and 34 min and were moderated by the first and third author, experienced in qualitative methods. All sessions were audio-recorded for analytical purposes, and deleted upon completion of the transcription process to ensure data protection and confidentiality.

### Interview Guide

An interview guide was developed in collaboration with educational experts and informed by relevant literature [20]; see Appendix 1. The guide included themes related to educational impact, user experience, and potential improvements to the VR technology. Follow-up questions were used to deepen and nuance the discussions.

The focus-group interviews aimed to elicit reflective responses from the students, with particular emphasis on the usability of the VR device and application, perceived transferability to clinical practice, and overall usefulness of VR as a preparatory tool for trauma simulation session. The participants were encouraged to suggest areas for improvement in the VR application.

### Data Analysis

The data were analyzed using qualitative content analysis, following established methodological principles [21, 22]. Audio recordings from the focus-group interviews were reviewed multiple times to ensure familiarity with the material. Meaning units were identified and manually extracted, then condensed and assigned descriptive codes. These codes were grouped into subcategories, then organized into overarching categories aligned with the study’s aim. Codes that did not correspond to the research objective were excluded from the final analysis.

The translation into English was undertaken by the second author in consensus with the other authors as part of the manuscript preparation. The results are presented in narrative form, structured according to the identified categories and subcategories. To enhance trustworthiness and transparency, representative quotations from participants are included to illustrate key findings.

### Ethics

The VRSinCT project was approved by the Swedish Ethical Review Authority on May 22, 2024 (Diary number: 2024-03341-01). All participants provided written informed consent to take part in the project and participate in the focus-group interviews. Prior to inclusion, the participants received detailed information about the study’s purpose, the voluntary nature of participation, and their right to withdraw at any time without consequences.

The study was conducted in accordance with the ethical principles outlined in the Declaration of Helsinki [23]. All data were handled in compliance with the General Data Protection Regulation (GDPR), and anonymization procedures were applied to ensure participant confidentiality and privacy.

## Results

The study participants were 16 female and 17 male students whose mean age was 26.3 (SD 4.11) years. The students’ prior experience with VR headsets varied from none to a few with relatively extensive gaming-related VR use. The results of the analysis were grouped into four categories and eleven subcategories (Table 1).

**Table 1:** Categories and subcategories

Categories	Subcategories
Perceived educational effect	• Motivational and demotivational impact • Knowledge transferability • Structure and design of the VR cases
Practical user experience	• Technical user engagement • Comfort-related issues
Comparison of VR and other educational forms	• VR as a useful tool • Advantages of other methods • Discrepancies between VR and CTC simulations
Areas for improvement in the Application	• Limitations and problems • Suggestions for improvement • VR’s future role in medical education

### Perceived Educational Effect

This category described both the benefits and limitations of using the VR application as preparation for non-VR trauma simulation session. While the application was generally perceived to be engaging and supportive of learning, several educational shortcomings were identified.

#### Motivational and Demotivational Impact

VR was perceived to be a stimulating and enjoyable learning method that supported engagement and focus, even when the participants were fatigued. The participants highlighted the interactive elements, such as scoring and feedback, as motivating features that encouraged preparation for the non-VR trauma simulation. Several noted that they would likely not have studied in advance without the VR application, and described it as a smooth way to combine theory and practice.

However, the experience was also described as repetitive and lacking in variety, which reduced engagement over time. The participants reported a decline in focus and motivation as the VR experience became repetitive and felt that the VR application did not increase their interest in the study material.

#### Knowledge Transferability

VR was generally perceived to be a useful introduction to clinical practice, allowing the participants to apply theoretical knowledge in a safe and structured environment. The visual and auditory realism of VR, provided by e.g. sound design and team communication, supported memorization and understanding of the ABCDE approach. The participants highlighted the value of structured roles, closed-loop communication, and team introductions, which they actively transferred to the trauma simulation. The VR application was also perceived as helping to reduce stress and increase confidence by helping them to become familiar with the physical environment and workflow at the CTC, contributing to a sense of preparedness and smoother team coordination.

Several participants expressed that the VR application created a false sense of preparedness for the non-VR trauma simulation session. The simplified scenarios and absence of complex clinical situations, such as compromised airways, contributed to uncertainty regarding roles and expected actions during the subsequent simulation. The participants stated that the training did not sufficiently reflect the complexity of real clinical environments, and that the time invested in the VR application was disproportionate to its educational value. As one participant noted, “the way it turned out now, that it ended up a bit off, and that maybe then /…/ maybe it [VR] is not better than no practice at all.”

#### Structure and Design of the VR Cases

The VR cases in the application were considered to be realistic, relevant, and well-structured, particularly for learning basic workflows. However, the lack of variety in terms of the cases was a recurring critique. Similar case openings caused confusion, and the short duration of the sessions reduced the educational value over time.

The participants found the response options to the multiple-choice questions in the VR application too easy and felt that many were obviously incorrect, which allowed them to rely on deduction rather than clinical reasoning. Over time they memorized the options, diminishing the need for critical thinking. Nonetheless, some appreciated the repetition of certain alternatives, describing them as “tricky” and useful for reinforcing appropriate decision-making.

### Practical User Experience

This category related to descriptions of the VR application as generally user-friendly and relevant for training, but also to several technical and comfort-related issues that limited usability and engagement.

#### Technical User Engagement

The VR application was perceived to be relatively intuitive, but technical challenges affected the overall experience. The participants disagreed on whether the instructions were sufficient, and several requested clearer guidance on how to use the controls, particularly the virtual hands and object interaction. A joint introduction to the equipment was proposed to improve usability.

Battery-related issues were frequently mentioned. Short battery life and difficulty locating the battery cover caused frustration and interrupted training. Bugs and glitches were also reported, including spontaneous teleportation, falling out of the simulation room, and incorrect scoring despite selecting the right answer.

The participants used the equipment both sitting and standing, but the choice did not influence the simulation outcome. Sitting down occasionally caused pixelation, and adjusting the VR goggles proved difficult, especially for those wearing glasses. Poor fit led to blurring and visual discomfort. The need for care when transporting and storing the expensive and fragile equipment was also considered to be a practical limitation.

#### Comfort-Related Issues

The use of the VR application was associated with some physical discomfort, including nausea, motion sickness, dizziness, and disorientation. These symptoms were typically transient, but in some cases impeded use of the equipment. Strategies such as sitting down, moving slowly, taking breaks, and closing the eyes were used to alleviate symptoms. Additional discomfort was reported from the headset itself, including forehead pressure, headaches, and eye fatigue, particularly due to the straps and fit of the goggles.

### Comparison of VR and Other Educational Forms

This category related to VR being seen as a valuable complement to traditional educational methods, particularly for translating theory into practice. However, the participants also identified several limitations compared to other learning formats, including discrepancies between VR and real-life simulations.

#### VR as a Useful Tool

VR was perceived to be a more engaging and personalized study method than purely theoretical approaches. It was felt to provide sensory stimulation and concrete examples of communication and workflow, which helped the participants to form mental representations of clinical practice. One participant reflected that VR resembled the structure of CTC scenarios, and allowed for individual practice: “To have the opportunity to do so alone without a complete group I believe is great /…/ it sticks so much better if I get to take my theoretical knowledge and convert it into practice.”

Despite these advantages, the participants noted that the VR application only allowed them to take on the role of a team leader and lacked tactile involvement and bedside procedures, making it less representative of real clinical work in other roles. While the equipment was mobile, it was considered cumbersome, and some felt that a large, clear space was required to use it. Concerns were raised about the feasibility of implementing VR on a larger scale, with some suggesting that it could be a waste of time and resources. At the same time, VR was seen as engaging and supportive of learning, and a potentially less resource-intensive alternative to physical simulations.

#### Advantages of Other Methods

The participants stated that reading was a more passive but time-efficient and relaxing form of learning than VR, and some preferred the former. The provided literature was considered to align better with CTC simulations and be more conducive to collaboration and peer learning. In contrast, the VR application was seen as more individualistic and less effective in promoting teamwork. It was suggested that a written test might have been as useful as the VR application, although others acknowledged that VR offered a more multi-dimensional understanding than text alone.

Clinical rotations were considered to be superior to VR for developing practical skills and competencies. Real-life settings were described as more unpredictable, complex, and emotionally demanding qualities deemed essential for learning. However, the limited responsibility given to students during rotations was problematized.

The CTC trauma simulation was viewed as more impactful and transferable to clinical practice than the VR application. The former was felt to encourage independent thinking and provide richer sensory input through physical interaction with mannequins. The participants stated that CTC trauma simulation should be prioritized, but acknowledged the constraints of resources and access. Regarding collaborative training, some noted that VR’s multiplayer potential could be replicated just as effectively with physical mannequins.

Participants perceived the multiple-choice format of the VR application as likely comparable to computer-based simulations, although they have not personally used such simulations. They assumed that a mobile or desktop version would be more accessible while offering similar educational value.

#### Discrepancies Between the VR Application and CTC Simulations

Although the VR and CTC cases were thematically aligned, the participants noted significant differences between the two in terms of structure and execution. VR simulations were felt to be more sequential and theory-driven, and CTC simulations were said to require simultaneous clinical decision-making and teamwork. This mismatch sometimes caused confusion and hindered performance. The participants reported feeling constrained by the VR structure, and experiencing improved cooperation once they abandoned it during the CTC simulation.

Differences in medical routines, such as naloxone dosing, and the inability to practise task distribution in VR led to a need to re-learn or adjust during the live simulation. One participant described the mismatch in leadership roles: “You were like a trauma leader in these [VR] scenarios /…/ you really stood on the side and directed… but that is not how it was at all now [at CTC]. You got a slightly distorted picture of that role.”

### Areas for Improvement in the VR Application

This category related to the participants viewing VR as a promising educational tool, particularly for safe and independent practice. However, several limitations of the current VR application were identified; concrete suggestions for improvement, as well as reflections on VR’s future role in medical education, were provided.

#### Limitations and Problems

The VR application was perceived to be overly rigid, limiting cognitive autonomy and clinical decision-making. The predetermined scenarios and multiple-choice format encouraged a “trial and error” approach rather than realistic reasoning. The participants felt this reduced the practical relevance of the training.

The application was also considered to be lacking in theoretical depth, hands-on elements, and interactivity. The graphical quality was described as insufficient, making it difficult to observe and learn from procedures. Features such as object retrieval in the trauma room were seen as non-contributory to learning. One participant summarized the experience as lacking realism and flexibility: “You just clicked through options /…/ it didn’t feel like real decision-making.”

#### Suggestions for Improvement

The participants emphasized the need for increased interactivity and responsiveness to enhance the realism of the VR application. They suggested enabling users to interact freely with avatars and equipment, assume different team roles, and perform clinical tasks such as examinations and interventions. Improved graphics and proximity to the patient were also proposed to support observational learning and build confidence.

The ability to learn from mistakes was highlighted as a key educational strength of VR. The participants preferred that simulations continue after incorrect decisions have been made, possibly with point deductions, to facilitate reflection and correction. One participant noted: “It would be better if the patient’s condition changed when you made a mistake—then you’d really have to think again.” They also recommended removing or complicating predefined response options to encourage deeper reasoning.

Feedback was appreciated in both its immediate and delayed forms within the simulation, either provided directly during the scenario or at its conclusion. The participants suggested linking feedback to relevant literature and including explanations to support understanding.

Greater control over the simulation—such as the ability to skip or revisit segments—was also requested to reduce waiting time and reinforce learning. However, some felt that waiting periods added realism and should not be removed entirely.

To increase authenticity, the participants proposed integrating real clinical cases into the application. A multi-player feature for group practice and teleportation for easier navigation was also suggested.

#### VR’s Future Role in Medical Education

While the participants were sceptical of VR as a standalone method, they saw potential in its use as a complementary tool in medical education. They suggested that VR could help bridge gaps in clinical rotations, and offer additional practice opportunities with fewer resources. VR was considered to be suitable for both pre-clinical and clinical students, particularly for preparation or repetition before Objective Structured Clinical Examinations (OSCEs) or surgical training.

To maximize its educational value, the participants emphasized the need for a clear theoretical foundation, and proposed integrating a study guide into the application, in order to ease the usability. Although VR was not seen as a replacement for practical training or internships, it was considered a possible alternative to SBME, provided a wider range of scenarios and greater complexity were included. A practical suggestion was to implement a booking system for VR use in designated rooms, such as at the CTC.

## Discussion

This study explored medical students’ experiences of using a VR application (TraumaSim) as preparation for non-VR trauma simulation. The findings indicate that VR has motivational potential and offers flexible, accessible learning opportunities, as a preparatory tool for SBME. However, in its current form, the TraumaSim application lacks the educational depth and realism required to replace established SBME methods.

One main result is that the VR application was perceived as an engaging and enjoyable learning method, promoting autonomous study and increasing motivation to prepare for non-VR trauma simulation. These findings align with previous research highlighting VR’s capacity to stimulate interest and enhance satisfaction in medical education [11, 13, 18]. The portability of VR technology also enables students to train independently and at their own pace, potentially increasing the number of practical study hours. However, comfort-related issues such as motion sickness, dizziness, and physical discomfort were reported, consistent with earlier studies [13, 18]. While these experiences were described as manageable by some participants, they were nonetheless perceived as limiting the overall usefulness of VR in its current preparatory format. Cyber sickness and physical discomfort are well recognized and partly inherent limitations of VR technology, and previous research suggests that such drawbacks may be tolerated when the perceived educational benefit clearly exceeds existing alternatives. This must be addressed before VR can be widely implemented in intensive educational programs such as medical school, as they may be influenced by variability across VR applications and software, which can contribute to cybersickness among students. Importantly, the variation in participants’ experiences reflects differing expectations, prior experiences, and learning preferences, rather than inconsistencies in the findings. Such divergence is a central result of this qualitative study and highlights how the perceived value of VR as a preparatory tool depends on contextual and individual factors.

Despite its motivational benefits, another main finding was that VR was not perceived as sufficient as a standalone learning modality. Participants emphasized that, in its current form, VR lacks the interactivity, responsiveness, and complexity required to support clinical reasoning and decision-making at a level comparable to SBME or clinical rotations. These findings are consistent with previous studies that question whether VR is ready to replace hands-on training [18, 24, 25]. The findings emphasize the importance of realism and cognitive autonomy, as the participants noted that the multiple-choice format and rigid structure hindered deeper learning. The need to “unlearn” VR routines during the CTC trauma simulation was seen as counterproductive, reinforcing the importance of aligning virtual training with clinical reality.

Another finding of this study is that several concrete areas for improvement were identified by the students to increase the educational value of the VR application. The results indicate the need for enhanced interactivity, dynamic patient responses, expanded roles, and more complex scenarios. The ability to learn from mistakes in a safe environment was highlighted as a key educational advantage, but the current design, where simulations ended after serious errors, was seen as limiting. Additionally, the limited number of available scenarios may have increased the risk of perceived repetitiveness, potentially leading to reduced motivation over time.

These insights support the notion that VR can complement traditional methods if applications are designed to reflect clinical complexity and allow for adaptive learninng and user autonomy [10, 11, 13, 25]. Suggestions such as booking systems and multiplayer modes reflect a desire to make VR more accessible and collaborative. When used alongside theoretical content and followed by non-VR trauma simulation, VR may support motivation, familiarization with workflows, and initial cognitive rehearsal prior to SBME. These findings appear to be related to the design and level of interactivity of the specific VR application [26], consistent with previous research showing that both perceptual factors, such as stereopsis, and pedagogical design choices influence the educational effectiveness of VR and related technologies [27, 28].

This study contributes a unique perspective by capturing student experiences directly linked to active education. As the primary stakeholders in medical training, students offer valuable insights into the educational utility and limitations of emerging technologies.

### Methodological Considerations

Reflexivity is central in qualitative research [29, 30]. The main analysis was conducted by a single researcher, which may have introduced interpretative bias. To strengthen credibility, the results were reviewed by the original interviewers at multiple stages, and consensus was reached through discussion. The analyst’s lack of involvement in data collection may have limited access to non-verbal cues and contextual nuances, but also reduced preconceptions and potential influence on interpretation.

While the sample size is appropriate for qualitative research, generalizability is limited. However, transferability is a more relevant marker of quality in this case [30, 31]. Importantly, the findings and identified limitations reflect the design and scope of the specific VR application and educational context studied, rather than inherent limitations of VR technology as such. The educational value of VR emerged as highly context-dependent, underscoring that its usefulness cannot be assumed but must be evaluated in relation to specific learners, learning objectives, and implementation contexts.

This qualitative study was not designed to evaluate the effectiveness of VR compared to traditional instruction, but to explore students’ experiences and perceptions of VR as a preparatory tool. Such experiential insights complement, rather than replace, outcome-focused randomized controlled studies.

### Conclusion and Future Directions

The findings suggest that, in its current form, the VR application was not perceived as a suitable standalone learning method. However, when used as a preparatory complement alongside theoretical content and followed by non-VR trauma simulation, VR may support motivation, early cognitive rehearsal, and orientation to simulation workflows.

Importantly, the identified limitations relate to the design and scope of the specific VR application studied and should not be interpreted as inherent limitations of VR technology in general.

Future research should build on these findings by evaluating how different VR designs and levels of complexity influence preparation for simulation-based learning, and by examining the educational impact of preparatory VR in combination with SBME using complementary qualitative and quantitative approaches.

## Appendix 1. Interview Guide (here translated to English)

This is an English translation of the original Swedish interview guide. Interviews were conducted in Swedish.

1. **How did you experience using VR goggles as part of the preparation for this course?**

- What was easy or difficult about the VR technology? Was there anything you particularly liked or disliked?
- How did VR affect your learning compared to other methods?

2. **What did you think about the cases in the VR training?**

- Which parts of the cases worked well or less well? Did you have different experiences of the cases?
- Is there anything you would like to change? Improve?

3. **Compared to only reading theoretical material, what did the VR training add?**

- Do you feel that VR gave you something you wouldn’t have gotten from just reading? Can you give some examples?
- How did VR change your understanding?

4. **After completing the simulation at KTC, how do you think the VR training may have influenced your performance in the simulation?**

- Was it helpful for your performance in the simulation? If so, how?
- Was there anything you missed in the VR training when you did the simulation?

5. **Do you think VR can replace parts of the practical training?**

- If yes, which parts? If no, what does practical simulation offer that VR cannot?
- How do you think VR can be used more in the future?

## Abbreviations

3D: Three-dimensional
ABCDE: Airway, Breathing, Circulation, Disability, Exposure
CTC: Clinical Training Centre
SBME: Simulation-based medical education
VR: Virtual reality
VRSinCT: Virtual-reality simulation in clinical teaching
OSCE: Objective structured clinical examination

## Declarations

### Use of Generative AI

Generative AI was not used in this study.
